# The Endonasal Endoscopic Approach to Different Sinonasal Fungal Balls

**DOI:** 10.1155/2022/6721896

**Published:** 2022-03-22

**Authors:** Ali Almomen, H Albaharna, Aishah A AlGhuneem, Batool Z AlZahir

**Affiliations:** ^1^King Fahad Specialist Hospital, Ministry of Health, Dammam, Eastern Province, Saudi Arabia; ^2^Qatif Central Hospital, MOH, Qatif, Saudi Arabia; ^3^Imam Abdulrahman Bin Faisal University, Dammam, Saudi Arabia; ^4^King Faisal University, AlAhsa'a, Hofuf, Saudi Arabia

## Abstract

**Background:**

Fungal ball sinusitis is a sinonasal fungus ball that usually affects immunocompetent adults with female predominance. The most affected sinus is the maxillary sinus. *Aspergillus* species is the most typically found fungus. Computed tomography (CT) scan is the gold standard tool in order to diagnose fungal ball sinusitis. The ultimate method for a fungal ball is functional endoscopic sinus surgery (FESS), which has a high success rate and a low morbidity rate.

**Objective:**

This study aims to demonstrate the various clinical presentations of fungal ball sinusitis including isolated maxillary sinus, sphenoid sinus, simultaneous occurrence of maxillary and sphenoid fungal ball, and post endonasal endoscopic pituitary surgery fungal ball with various age groups. Also, this study aims to emphasize the importance of early diagnosis and treatment in such cases. *Patients and Methods*. A retrospective study that was carried in the otorhinolaryngology department of two hospitals: King Fahad Specialist Hospital and Qatif Central Hospital, Eastern Region, Saudi Arabia. The study was conducted on a total of 16 patients who were diagnosed with paranasal sinuses fungal ball in an 11-year period from January 2008 and November 2019.

**Results:**

Out of 16 patients with paranasal sinuses fungal ball, 11 cases were female and 5 males, with age ranging between 16 and 46 years. Results showed eight isolated sphenoid (50%), six isolated maxillary fungal ball (38%), one simultaneous occurrence of the sphenoid and maxillary fungal ball (6%), and one post endonasal endoscopic pituitary surgery for pituitary adenoma (6%). CT scan was performed for all 16 cases which is the standard tool for the diagnosis of the fungal ball.

**Conclusion:**

Fungal ball may present with variety of symptoms but most commonly with postnasal discharge (PND), headache, and facial pain. CT sinuses is the diagnostic radiological modality to confirm the diagnosis. The FESS functional endoscopic sinus surgery is the gold safe approach for patients with fungal ball to manage their symptoms, confirm the diagnosis, and removal of disease with no morbidities.

## 1. Introduction 

The most common form of fungal rhinosinusitis is sinonasal fungus ball which usually presents in adults with normal immunity where maxillary sinus being the most involved site [1, 2]. Previously, it was termed aspergillomas according to the most commonly detected fungus being *Aspergillus* species [3]. Histologically, fungal ball is an accumulation of dense matter of fungal hyphae without invasion features [2]. The pathogenesis of fungal ball formation is still unclear. Moreover, there is female predominance and those of older age as demonstrated in previous studies [4]. Patients usually present with nasal obstruction, facial pain, postnasal drip, and purulent discharge; however, others may be asymptomatic [5]. Findings on endoscopic examination of the nasal cavity range from normal mucosa and nasal cavity to crusting edematous mucosa with polyp formation and purulent discharge [6]. CT scans of paranasal sinuses are commonly performed [1, 7]. The main purpose of this study is to illustrate various presentations of paranasal sinuses fungal ball and highlighting the importance of appropriate diagnosis and treatment in such cases.

## 2. Method

An 11-year retrospective study was conducted on 16 patients with paranasal sinuses fungal ball from January 2008 and November 2019. The study was conducted at the otorhinolaryngology department at two hospitals: King Fahad Specialist Hospital and Qatif Central Hospital, Eastern Region, Saudi Arabia. Clinical presentation, CT scan, endoscopic examination, fungal culture, and surgical treatment were studied and reviewed.

### 2.1. IRB

This clinical research was approved by the Institutional Review Board (IRB) at King Fahad Specialist Hospital, Dammam, KSA.

## 3. Results

**Figure 1:**
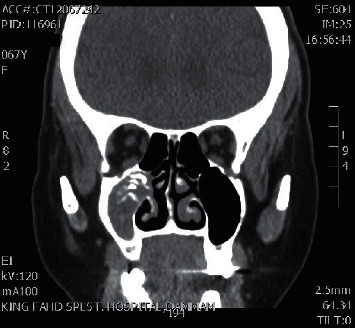
Coronal CT sinuses showing right maxillary sinus fungal ball.

**Figure 2:**
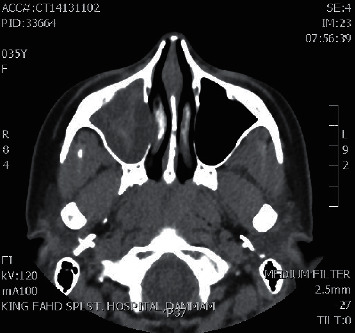
Axial CT sinuses showing right maxillary sinus fungal ball.

**Figure 3:**
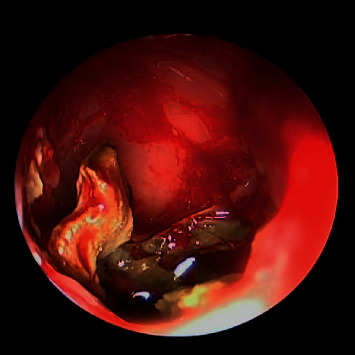
30-degree endoscopic view showing right maxillary fungal ball.

**Figure 4:**
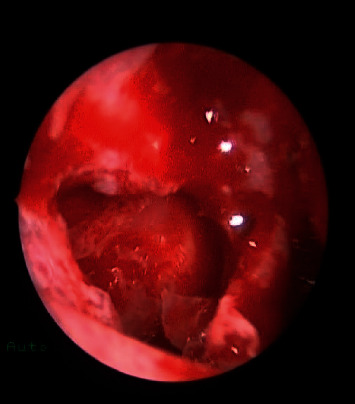
Postoperative endoscopic view showing wide middle meatus antrostomy with patent maxillary sinus.

**Figure 5:**
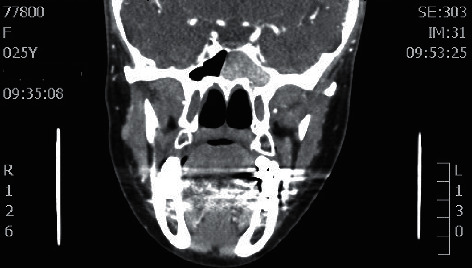
Coronal CT sinuses showing left sphenoid sinus fungal ball.

**Figure 6:**
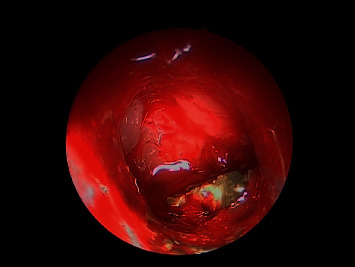
Intraoperative endoscopic view showing left sphenoid sinus fungal ball.

**Figure 7:**
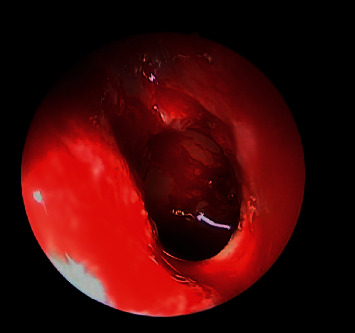
Postoperative endoscopic view showing wide sphenoidectomy post removal of fungal debris.

**Figure 8:**
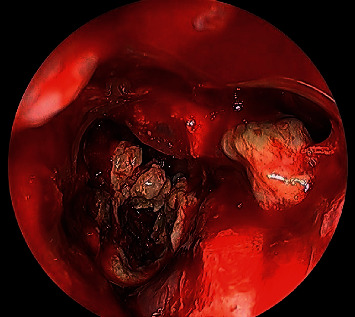
0-degree intraoperative endoscopic view fungal balls filling both left maxillary and sphenoid sinuses.

**Figure 9:**
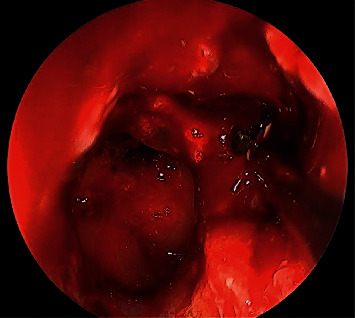
Postoperative debridement and complete removal of fungal balls from both left maxillary and sphenoid sinuses.

**Figure 10:**
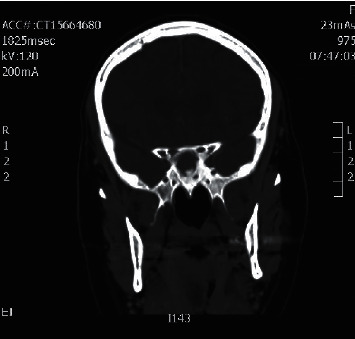
Coronal CT scan sinuses showing a sphenoid opacity of fungal ball post endonasal endoscopic pituitary surgery.

**Figure 11:**
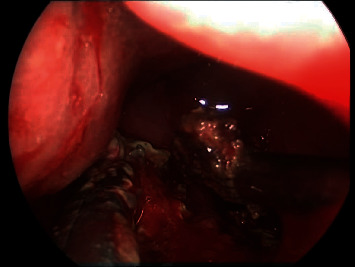
Intraoperative endoscopic view showing the sphenoid is full with fungal debris post endonasal endoscopic pituitary surgery.

**Figure 12:**
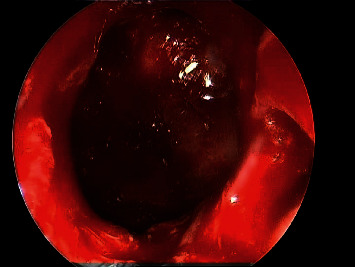
Postoperative endoscopic view showing a wide clean sphenoid sinus.

A total of 16 patients were diagnosed with paranasal sinuses fungal ball. Cases included 6 isolated maxillary fungal ball (Figures 1–4), 8 isolated sphenoid (Figures 5–7), one simultaneous occurrence of sphenoid and maxillary fungal ball (Figures 8–9), and one post endonasal endoscopic pituitary surgery for pituitary adenoma (Figures 10–12). Moreover, female patients represented 11 cases and the other 5 cases were male patients as the age ranged between 16 and 46 years. The most common symptoms were postnasal discharge (PND), headache, and facial pain, consecutively. Computed tomography (CT) sinuses is the radiological modality for work up and diagnosis. However, the final diagnosis was confirmed intraoperatively or by fungal culture. Two out of the sixteen cases were positive for fungal cultures as the most common organism was *Aspergillus*. All cases were successfully managed by the endonasal endoscopic approach with no preoperative nor postoperative complications.

Table 1 is the summary of the studied patients.

### 3.1. Illustrative Cases

#### 3.1.1. Maxillary Sinus Fungal Ball should include figure 1–4

#### 3.1.2. Sphenoid Sinus Fungal Ball should include figure 5–7

#### 3.1.3. Simultaneous Occurence of Sphenoid and Maxillary should include figure 8–9

#### 3.1.4. Post Endonasal Endoscopic Pituitary Surgery should include figure 10–12

## 4. Discussion

Fungal rhinosinusitis (FRS) is commonly classified into invasive and noninvasive fungal rhinosinusitis based on histological tissue invasion by fungi. The invasive disease is further classified into acute invasive (fulminant) FRS, granulomatous invasive FRS, and chronic invasive FRS. On the other hand, noninvasive fungal sinusitis which is clinically behaving like chronic bacterial sinusitis. It includes 3 type saprophytic fungal infestation, fungal ball, and fungus-related eosinophilic rhinosinusitis including allergic fungal rhinosinusitis (AFRS) [8–10]. Fungal ball is chronic noninvasive accumulation and dense conglomeration fungal hyphae found in paranasal sinuses [8, 11]. It is important to differentiate between allergic fungal rhinosinusitis and fungal ball. AFRS is diffuse, involves bilateral sinuses, and is usually associated with nasal polyps. On the other hand, fungal ball presents unilaterally, and maxillary sinus is most affected [12]. It is predominantly seen in immunocompetent elderly females with an average age at presentation being 64 (range 14–90) years old [8, 10, 13]. In this study, the majority of cases were females with age ranging between 16 and 46 years. Among all sinuses, the maxillary sinus is the most common site of occurrence (94%) followed by sphenoidal sinus (4–8%). The ethmoid (3%) and frontal sinus (2%) rarely occur [14]. In our study, there were six cases of maxillary, eight cases of sphenoid, one case of post endonasal endoscopic pituitary surgery, and the first reported case of simultaneous occurrence of maxillary and sphenoid sinus fungal ball. Furthermore, there is a female predominance (2 : 1) ratio, as reported in our study; eleven of the cases were female, with the remaining five cases being male.

Regarding the risk factors for developing a fungal ball are mainly unclear. A case-control study demonstrated the association of maxillary fungus in patients who underwent endodontic sealers, with a rate as high as 89.2% in those patients. This is due to certain ingredients of sealers such as zinc oxide that may facilitate fungal growth. Moreover, the risk of maxillary fungal ball is fourteen times compared with patients without endodontic maxillary teeth treatment [6].

Patients with fungal balls typically present with nonspecific symptoms with various rhinological complaints that develop slowly and asymptomatically, which explains the late presentation of those cases. These symptoms vary depending on the involved sinus. In maxillary fungal ball, patients report facial pain and/or pressure, purulent rhinorrhea, nasal obstruction, and olfactory dysfunction. In our study, patients with maxillary sinus fungal ball, postnasal discharge, and facial pain were the most common complaints [8, 9, 11, 13, 14].

Headache, postnasal discharge, and cough are commonly observed in sphenoid fungal ball. Other symptoms include chronic cough, olfactory dysfunction, and cacosmia. Less common symptoms include convulsions, epistaxis, proptosis, fever, cough, and blurred vision. However, some patients may be asymptomatic [8, 9, 12, 15, 16].

The most common causative organism of FB is the *Aspergillus* species [3, 8, 9, 11]. A study was conducted on one hundred and seven patients with fungal ball who had fungal culture; fifty-one percent of them had positive fungal cultures with *Aspergillus* sp. as it appears to be the most frequent organism [17]. In our study, two fungal ball cases showed positive culture for *Aspergillus* sp. as for the remaining cases were negative culture.

A fungal ball is diagnosed according to radiological and histopathological findings, as DeShazo et al. proposed [7] (see Table 2).

Definitive diagnosis of a fungal ball can be achieved by distinctive histopathology of the twisted fungal hyphae. Moreover, a CT scan is the imaging of choice for diagnosing those patients. It is significant for determining surgical landmarks for the endoscopic approach and the extent of invasion of the disease. Almost 99% of cases are unilateral with single sinus involvement seen in 94%. Bilateral involvement and multiple sinuses may exist. A retrospective study was done, selecting 81 patients who met DeShazo's criteria, imaging studies were done, CT evaluation revealed bone erosion in twenty-seven cases [14]. Another retrospective study included 43 patients with unilateral maxillary fungal ball who underwent endoscopic sinus surgery. Using CT scan, evaluation of bony changes and a wall thickness comparison of diseased sinus with normal sinus were applied. Results showed that 95% of these patients had evident osteitic changes in the anterior, lateral, and posterior walls of the involved sinus. The medial wall of the involved maxillary sinus was not evaluated because the majority of cases illustrated evident erosion of the medial wall [18]. In addition, in some cases, MRI is used for diagnosing fungal ball with bony lysis and possible orbital or brain connection [8, 9, 16].

Untreated fungal of paranasal sinuses can result in a variety of complications. Recurrent bacterial sinusitis is the most frequent complication where the fungal material acts as a foreign body. Mucoceles and pyoceles have also been reported. Neurological complications including optic neuritis, ophthalmoplegia and seizure have also been rarely described. If a patient becomes immunodeficient, noninvasive colonization may transform into invasive fungal infection [8]. In our study, fortunately, no preoperative or postoperative complications were observed.

Surgical treatment is indicated when the patient is symptomatic and apparent sinus opacification on imaging studies. The aim of surgical treatment is to achieve a complete removal of fungal ball to restore ventilation. Functional endoscopic sinus surgery (FESS) is the definitive approach for fungal ball with high success and low morbidity rates [8, 9, 11]. There are different methods used depending on the affected sinus. In a retrospective study, a total of 81 patients diagnosed with fungal sinusitis, all patients underwent functional endoscopic sinus surgery. While in patients with maxillary and ethmoidal sinusitis, meatotomy and ethmoidectomy were performed. Out of 50 patients with maxillary fungus balls, 30 patients (66%) underwent sinusoscopy via fossa canina. Twenty patients had sphenoid fungus ball. Half of the patients had a transnasal sphenoidotomy and another half had a transethmoidal approach [14]. Another retrospective study was conducted at Chonnam National University Hospital and Hwasun Hospital, in which 245 patients were diagnosed with fungal ball sinusitis. All patients were treated surgically by endoscopic sinus surgery. A middle meatal antrostomy and removal of the hyphal mass were performed in a maxillary fungus ball. A transnasal sphenoidotomy was performed on the sphenoid fungus ball [20].

In our study, out of 16, there were 8 cases presented with isolated sphenoid fungus ball. All cases were managed by the direct transnasal sphenoidotomy approach. There were 6 cases of isolated maxillary fungus ball, and uncinectomy and wide adequate maxillary antrostomy were performed using the 0-degree, 30-degree, and 45-degree angled endoscopes.

The extent of the surgical procedure is congruent with the preoperative imaging studies and the intraoperative findings. Classically, a wide opening of the involved sinus or sinuses and complete removal of all the fungal concrements is required with the use of curved suctions and forceps. Cautious must be taken when a dental filling is present within the sinus, thus, it should be thoroughly evacuated to avoid acting as a nidus for regrowth of the fungus ball. A biopsy from the mucosa must be taken to exclude any microscopic invasion by fungus. Some authors recommended rinsing the cavity with normal saline or with an iodine solution [8, 18]. In order to achieve complete cleaning of sinuses, patients were advised to use nasal saline irrigation. Additionally, sinus cavity debridement, topical nasal steroids, and a short-course antibiotic were all used as postoperative care after FESS [19].

In our experience, all the cases were successfully managed with no complications reported by the endonasal endoscopic approach using the 0-degree, 30-degree, and 45-degree angled endoscopes to visualize the sinus recesses and the lateral extension of sphenoid sinus for the fungal removal and rinsing of the involved sinuses.

Recurrent or persistent disease is most often detected during the first 2- or 4-years postsurgery investigations where the patient may present with postnasal discharge. A minimally invasive surgical procedure (reopening of the sinusotomy, suctioning, and washing of the fungal concrements) aims for resolution of the fungal ball. Follow-up for such cases both clinically and endoscopically is essential specially for patients with recurrent or persistent disease who have symptoms or abnormal nasal endoscopic findings [8, 18]. In our study, no residual nor recurrent cases were reported with no morbidities related to the procedure with a clinical follow-up of 8 years post treatment.

## 5. Conclusion

In summary, appropriate diagnosis of fungal ball is crucial where specific investigations should be performed. CT scan of the PNS and nasal endoscopy with pathology are useful and highly specific in reaching the diagnosis. The most common presenting symptoms were postnasal discharge (PND), headache, and facial pain. Functional endoscopic sinus surgery is the gold safe standard approach for patients with fungal ball aiming for fungal removal and sinus wash outs.

## Figures and Tables

**Table 1:** Summarizes the total of 16 patients included in the study illustrating their characteristics, presenting symptoms, and fungal ball culture results.

Patient no.	Sex	Age	Presenting symptoms	Diagnosis	Fungal culture result
Maxillary fungal ball					
1	Female	33	Facial pain and postnasal discharge	Fungal ball	−ve culture
2	Female	40	Postnasal discharge and cough		−ve culture
3	Male	35	Right facial fullness		−ve culture
4	Female	40	Facial pressure and postnasal discharge		−ve culture
5	Female	35	Postnasal discharge and cough		−+ve *Aspergillum*
6	Female	45	Facial pain		−ve culture

Sphenoid fungal ball					
7	Male	43	Postnasal discharge	Fungal ball	+ve *Aspergillum*
8	Female	31	Postnasal discharge		−ve culture
9	Male	39	Headache and postnasal discharge		−ve culture
10	Female	37	Cough and postnasal discharge		−ve culture
11	Female	35	Headache and postnasal discharge		−ve culture
12	Male	14	Asymptomatic		−ve culture
13	Female	46	Headache and postnasal discharge		−ve culture
14	Male	35	Headache		−ve culture

Simultaneous occurrence of maxillary and sphenoid fungal ball					
15	Female	52	Headache, facial pain, and postnasal discharge	Fungal ball	−ve culture

Post endonasal endoscopic pituitary surgery for pituitary adenoma					
16	Female	35	Occipital headache	Fungal ball	−ve culture

**Table 2:** DeShazo et al.'s [7] diagnostic criteria of fungus ball.


1. Radiological evidence of sinus opacification with or without associated flocculent calcifications
2. Mucopurulent, cheesy, or clay-like material within a sinus
3. A matted, dense conglomeration of hyphae separate from but adjacent to sinus respiratory mucosa
4. A chronic inflammatory response of variable intensity in the mucosa adjacent to fungal elements. This response includes lymphocytes, plasma cells, mat cells, and eosinophils without an eosinophil predominance or a granuloma response. Allergic mucin is absent on hematoxylin-eosin-stained material
5. No histological evidence of fungal invasion of mucosa, associated blood vessels, or underlying bone visualized microscopically on gomori methenamine silver or other special stains for fungus

## Data Availability

All data described in the study are included in this published article.

## Abbreviations:

FESS: Functional endoscopic sinus surgery
CT: Computed tomography
PND: Postnasal discharge
FRS: Fungal rhinosinusitis
AFRS: Allergic fungal rhinosinusitis.

## References

[1] Dufour X., Kauffmann-Lacroix C., Ferrie J. C., Goujon J. M., Rodier M. H., Klossek J. M. (2006). Paranasal sinus fungus ball: epidemiology, clinical features and diagnosis. A retrospective analysis of 173 cases from a single medical center in France 1989–2002. *Sabouraudia*.

[2] Jiang R. S., Huang W. C., Liang K. L. (2018). Characteristics of sinus fungus ball: a unique form of rhinosinusitis. *Clinical Medicine Insights: Ear, Nose and Throat*.

[3] Montone K. T. (2016). Pathology of fungal rhinosinusitis: a review. *Head and neck pathology*.

[4] Yoon Y. H., Xu J., Park S. K., Heo J. H., Kim Y. M., Rha K. S. (2017). A retrospective analysis of 538 sinonasal fungus ball cases treated at a single tertiary medical center in Korea (1996–2015). *International forum of allergy & rhinology*.

[5] Nomura K., Asaka D., Nakayama T. (2013). Sinus fungus ball in the Japanese population: clinical and imaging characteristics of 104 cases. *International Journal of Otolaryngology*.

[6] Deutsch P. G., Whittaker J., Prasad S. (2019). Invasive and non-invasive fungal rhinosinusitis-A review and update of the evidence. *Medicina*.

[7] DeShazo R., Obrien M., Chapin K. (1997). Criteria for the diagnosis of sinus mycetoma. *The Journal of Allergy and Clinical Immunology*.

[8] Grosjean P., Weber R. (2007). Fungus balls of the paranasal sinuses: a review. *European Archives of Oto-Rhino-Laryngology*.

[9] DeShazo R. D., Chapin K., Swain R. E. (1997). Fungal sinusitis. *New England Journal of Medicine*.

[10] Chakrabarti A., Denning D. W., Ferguson B. J. (2009). Fungal rhinosinusitis. *The Laryngoscope*.

[11] Seo M. Y., Lee S. H., Ryu G. (2019). Clinical pattern of fungal balls in the paranasal sinuses: our experience with 70 patients. *European Archives of Oto-Rhino-Laryngology*.

[12] Lee J.-H., Lee B.-D. (2020). Characteristic features of fungus ball in the maxillary sinus and the location of intralesional calcifications on computed tomographic images: a report of 2 cases. *Imaging Science in Dentistry*.

[13] Agarwal S. (2010). Paranasal sinuses fungal balls in children. *Children*.

[14] Pagella F., Matti E., Bernardi F. D. (2007). Paranasal sinus fungus ball: diagnosis and management. *Mycoses*.

[15] Toplu Y. (2018). Paranasal sınus fungus balls: our experıence of 19 cases and Revıew of lıterature. *Medical Research Archives*.

[16] Shcherbakov D., Klimova N., Malysheva T., Shcherbakova A. (2019). Large fungal ball of the paranasal sinuses and nasal cavity: two case reports. *International Journal of Biomedicine*.

[17] Montone K. T., Livolsi V. A., Feldman M. D. (2012). Fungal rhinosinusitis: a retrospective microbiologic and pathologic review of 400 patients at a single university medical center. *International Journal of Otolaryngology*.

[18] Klossek J.-M., Serrano E., Péloquin L., Percodani J., Fontanel J.-P., Pessey J.-J. (1997). Functional endoscopic sinus surgery and 109 mycetomas of paranasal sinuses. *The Laryngoscope*.

[19] Jun Y. J., Shin J. M., Lee J. Y., Baek B. J. (2018). Bony changes in a unilateral maxillary sinus fungal ball. *Journal of Craniofacial Surgery*.

[20] Lee D. H., Joo Y. E., Lim S. C. (2013). Fungus balls of the bilateral paranasal sinuses. *Indian Journal of Otolaryngology and Head & Neck Surgery*.

